# High-Throughput Sequencing of Microbial Community Diversity and Dynamics during Douchi Fermentation

**DOI:** 10.1371/journal.pone.0168166

**Published:** 2016-12-19

**Authors:** Lin Yang, Hui-lin Yang, Zong-cai Tu, Xiao-lan Wang

**Affiliations:** Key Lab of Protection and Utilization of Subtropic Plant Resources of Jiangxi Province, Jiangxi Normal University, Nanchang, China; University of Naples Federico II, ITALY

## Abstract

Douchi is a type of Chinese traditional fermented food that is an important source of protein and is used in flavouring ingredients. The end product is affected by the microbial community present during fermentation, but exactly how microbes influence the fermentation process remains poorly understood. We used an Illumina MiSeq approach to investigate bacterial and fungal community diversity during both douchi-koji making and fermentation. A total of 181,443 high quality bacterial 16S rRNA sequences and 221,059 high quality fungal internal transcribed spacer reads were used for taxonomic classification, revealing eight bacterial and three fungal phyla. *Firmicutes*, *Actinobacteria* and *Proteobacteria* were the dominant bacterial phyla, while *Ascomycota* and *Zygomycota* were the dominant fungal phyla. At the genus level, *Staphylococcus* and *Weissella* were the dominant bacteria, while *Aspergillus* and *Lichtheimia* were the dominant fungi. Principal coordinate analysis showed structural separation between the composition of bacteria in koji making and fermentation. However, multivariate analysis of variance based on unweighted UniFrac distances did identify distinct differences (p <0.05), and redundancy analysis identified two key genera that are largely responsible for the differences in bacterial composition between the two steps. *Staphylococcus* was enriched in koji making, while *Corynebacterium* was enriched in fermentation. This is the first investigation to integrate douchi fermentation and koji making and fermentation processes through this technological approach. The results provide insight into the microbiome of the douchi fermentation process, and reveal a structural separation that may be stratified by the environment during the production of this traditional fermented food.

## Introduction

Douchi is a fermented food that is important for flavouring food and that has been produced in China and several other countries for thousands of years [1]. Traditional Chinese medicine also uses douchi for treating dyspepsia, restlessness, asthma, and to increase sweating [2,3]. Recent studies indicated that douchi can inhibit prostate and breast cancer [4], and it also displays anti-diabetic activity [5] and protects against osteoporosis and cardiovascular diseases [6].

Douchi is produced through the fermentation of soybean by naturally occurring microorganisms. In general, douchi is produced in two stages; an initial koji making stage, and a subsequent fermentation stage. To make douchi, black beans used as the raw material are screened, steamed at 115°C for 30 min to soften, cooled, and treated with the ‘house flora’ that initiates koji making. At this stage, a koji inoculum (5%) from a later stage of production is inoculated into the black beans and koji making is continued for 7 days until the beans are covered with a white mold. Room temperature is maintained throughout koji making, and koji is washed in order to remove mycelia and spores that could otherwise make the douchi taste bitter [7]. The black beans are then mixed with salt (5% w/w) to prevent the growth of microorganisms and transferred to fermentation tanks which are sealed to exclude oxygen. The fermentation stage lasts for 15 days at a temperature of ~55°C, and the final product is dried in an oven. During the koji making stage, *Aspergillus oryzaethe* is traditionally used for inoculation, while other microorganisms such as *Bacillus* spp. are used to produce proteases for the degradation of peptides in the subsequent fermentation stage. During koji making, these microorganisms are a source of various enzymes and metabolites for fermentation, including glutaminase, cellulose, proteases, lipases, amylases and fibrinolytic enzymes, that are needed for the degradation of key ingredients such as lipids, proteins, carbohydrates and other functional constituents [8]. Fermentation is then performed in fermentation tanks, and this stage creates the characteristic flavours and nutritional content. Since douchi fermentation is performed in a relatively uncontrolled and spontaneous manner, the results can be inconsistent, and a better understanding of the microbial community diversity during fermentation would be useful for ensuring adequate quality of the final product.

The influence of the molecular ecology and culturing method on the diversity, composition and dynamics of the microbial community have been investigated using cell cultures, colony counting, denaturing gradient gel electrophoresis [9,10], temperature gradient gel electrophoresis [11–13], microarray [14] and length heterogeneity polymerase chain reaction [15,16]. However, a complete understanding of the microbial community remains elusive [17]. Illumina MiSeq has been used to investigate many microbial ecosystems, including milk [17,18], landfill leachate treatment [19], Italian salami [20], gut [21], and cheese [22–24]. Such high-throughput sequencing approaches can provide a more comprehensive insight into microbial community diversity without the bias associated with some of the other techniques [25]. Furthermore, Illumina MiSeq has the benefit of easy miniaturization and parallelization, which dramatically decreases the cost of microbial community diversity analysis.

Although high-throughput sequencing is now widely used, until now this approach has not been employed to study microbial community diversity in the douchi fermentation process. In the present study, we used Illumina MiSeq to study microbial community diversity in the douchi fermentation process. The results should enhance our understanding of the microbiome in this traditional fermented food.

## Materials and Methods

### Sampling

A total of 10 samples were included in this study. Samples were collected from koji making and fermentation in the workshop of Daoxiangyuan corporation (Jiangxi province, China). All samples were taken from the same site. Samples were taken from koji making on day 1, 3, 5, and 7, and from fermentation on day 1, 3, 6, 9, 12, and 15. Samples (100 g) were collected in triplicate, mixed together to reduce errors and immediately stored at −80°C for subsequent analysis.

### DNA extraction

DNA was extracted during koji making and fermentation with an OMEGA E.Z.N.A soil DNA kit purchased from Feiyang BIOTECH Co., Ltd. (Guangzhou, China) according to the manufacturer’s instructions without modifications [26]. DNA quality was monitored by 0.8% agarose gel electrophoresis, and DNA was stored at −80°C for further analysis.

### Illumina MiSeq sequencing

The V4 region of the 16S rRNA was amplified using primers 515F (5′-GTGCCAGCMGCCGCGGTAA-3′) and 806R (5′-GGACTACHVGGGTWTCTAAT-3′), and the internal transcribed spacer 1 (ITS 1) region of fungi amplicons was amplified with forward (5′-CTTGGTCATTTAGAGGAAGTAA-3′) and reverse (5′-GCTGCGTTCTTCATCGATGC-3′) primers. 5′-barcoded amplicons were generated using Ex Taq HS (TaKaRa Bio Inc., Shiga, Japan) by PCR under conditions of 2 min at 95°C, followed by 35 cycles at 95°C for 30 s, annealing at 55°C for 1 min, extension at 72°C for 1 min, and a final extension at 72°C for 10 min. Amplicons were pooled in equimolar concentrations and sequenced by an Illumina MiSeq platform and MiSeq Reagent Kit v1 (Illumina, Inc., Santiago, CA, USA) at the Beijing Genomics Institute (Shenzhen, China).

Clean data were obtained using scripts written in house as follows: (1) removal of sequences containing more than one ambiguous base (N); (2) confirmation of barcode and adaptor completeness; (3) removal of sequences shorter than 100 bp. All 250 bp pair-end sequence reads were connected using COPE software (Connecting Overlapped Pair-end, V 1.2.1) [27] to merge read pairs into tags from DNA fragments. Further data processing was performed as previously described [28–32,18] and included removal of sequencing noise using the pre.cluster tool in the MOTHUR software package v. 1.31.2 [33], and *de novo* chimera detection and removal in UCHIME v. 4.2. Operational taxonomic units (OTUs) were also determined using MOTHUR, with a 97% sequence identity threshold [33]. 16S rRNA reads were assigned using 16S rRNA training set 9 in the RDP database using a local BLAST search. A local BLAST search was also used to assign ITS 1 reads with the NCBI GenBank database (for all ITS 1 sequences with taxonomic annotations).

### Statistical analysis

Alpha diversity including Chao 1, Simpson diversity and Shannon diversity indices, as well as observed species and phylogenetic diversity [34], were subjected to statistical analysis using the MOTHUR package. Principal coordinate analysis (PCoA), which was used to measure dissimilarity at phylogenetic distances based on UniFrac analysis, was performed with QIIME and visualized using KING [35]. Statistically significant differences between koji making and fermentation were determined using the non-parametric Mann—Whitney test and multivariate analysis of variance in MATLAB R2014a (The Math works, Natick, MA, USA) and Canoco for Windows 4.5 (Microcomputer Power, NY, USA).

### Nucleotide sequence accession numbers

Sequences reported in this paper are available in the SRA database under accession numbers SRX1925639–SRX1925640, SRX1925651–SRX1925658 (bacterial 16S rRNA gene sequences) and SRX1925641–SRX1925650 (fungi ITS 1 region sequences).

## Results and Discussion

### Abundance and diversity of members of the bacterial and fungal microbiota

Illumina MiSeq sequencing generated 181,443 high quality bacterial tags from 10 examined sample sets, with an average of 18,144 tags per sample (range = 15,944–19,846, SD = 1,246). Meanwhile, a total of 221,059 high quality ITS tags were obtained from koji making and fermentation across the entire douchi fermentation process. Sequencing results are shown in Table 1 High quality sequences were grouped into 1083 OTUs for bacteria and 88 OTUs for fungi (both at the 97% similarity level), and after removing singletons, the average number of OTUs was 115 for bacteria (range = 66–167, SD = 39.19) and 22 for fungi (range = 12–44, SD = 9), and these were subjected to further analysis.

**Table 1:** Sample information, microbial diversity and sequence abundance.

Sample	Number of reads		Number of OTUs		Shannon index		Simpson index		Chao 1 index		Observed species	
	Bacteria	Fungi	Bacteria	Fungi	Bacteria	Fungi	Bacteria	Fungi	Bacteria	Fungi	Bacteria	Fungi
Koji making 1	17092	21913	73	30	1.05	1.08	0.5	0.45	199.43	98	101	30
Koji making 3	17809	21469	49	12	0.82	0.92	0.49	0.54	96	12	65	12
Koji making 5	18797	20365	66	13	0.84	0.75	0.59	0.69	240.13	14	87	13
Koji making 7	16509	22484	121	44	1.47	0.57	0.39	0.75	476.79	86.85714	200	44
Mean ± SD	17551.75 ± 985.82	21557.75 ± 897.16	77.25 ± 30.86	24.75 ± 15.26	1.04 ± 0.30	0.83 ± 0.22	0.49 ± 0.08	0.61 ± 0.14	253.08 ± 161.00	52.71 ± 46.09	113.25 ± 59.70	24.75 ± 15.26
Fermentation 1	15994	20900	141	15	1.64	0.7	0.27	0.7	461.57	16.5	222	15
Fermentation 3	17731	22727	128	15	1.58	0.56	0.32	0.76	424.29	15.6	188	15
Fermentation 6	19848	23100	118	17	1.35	1.02	0.36	0.43	432.11	20	161	17
Fermentation 9	19047	23348	150	23	1.48	1.05	0.41	0.43	526.62	30	214	23
Fermentation 12	19323	22510	137	22	1.87	1.04	0.26	0.45	530.92	32.5	208	22
Fermentation 15	19293	21457	167	32	2.75	0.97	0.11	0.45	1299.78	67	367	32
Mean ± SD	18539.33 ± 1434.20	22340.33 ± 961.83	140.17 ± 17.13	20.67 ± 6.53	1.78 ± 0.51	0.89 ± 0.21	0.29 ± 0.10	0.54 ± 0.15	612.55 ± 339.74	30.27 ± 19.31	226.67 ± 72.15	20.67 ± 6.53

Rarefaction curves for (a) observed species and (b) Simpson diversity indices for bacteria and fungi are shown in S1 and S2 Figs, respectively. Although the rarefaction cure is not parallel with the x-axis, the Simpson diversity index reached saturation, suggesting that some additional phenotypes could be added with additional sequencing, but the great majority of microbial diversity was captured. Meanwhile, the Shannon diversity, Chao 1 and observed species indices were applied to measure bacterial and fungal sequence abundance and diversity during douchi fermentation.

Diversity indices of koji making and fermentation (S1 and S2 Tables) indicated significant differences in bacterial diversity between steps (p <0.05). In contrast, no significant differences were apparent in fungal diversity (p > 0.05).

Diversity indices indicated that the diversity of the bacterial community increased as the koji making stage progressed, consistent with a previous report [25]. However, bacterial diversity then declined during the early stages of fermentation, before gradually recovering. This suggests that bacteria were prolific during koji making, but suffered when transferred to the fermentation environment, presumably due to the high temperature, low humidity, oxygen scarcity, and high salt content [36]. These harsh conditions likely inhibited the bacteria until they adapted to, and became tolerant of, the harsh fermentation environment. Temperature, salt, humidity and submerged fermentation can all inhibit or kill microorganisms that are not tolerant to these environmental challenges, unlike the halotolerant and anaerobic microbes that can thrive during the product-making process [25]. Alternatively, interspecific competition might be a factor causing the decrease in diversity indices, and bacteria that can adapt to the fermentation environment survive and eventually thrive. In the present study, fungal diversity remained stable during both koji making and fermentation, suggesting the fungal species adapted to both environments, and could tolerate the transition between steps.

### Comparison of bacterial communities in koji making and fermentation

**Fig 1:**
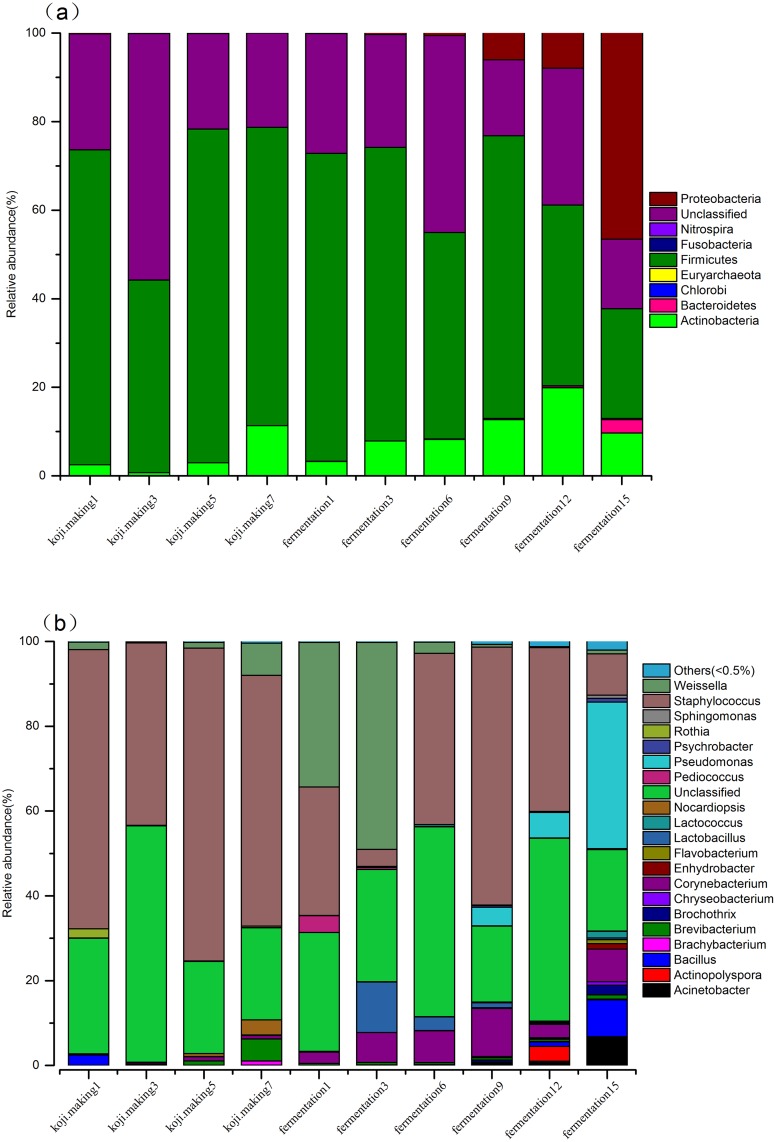
Relative abundance of bacteria at phyla (a) and genus (b) levels during koji making and fermentation.

Eight bacterial phyla were identified in the koji making step, consisting of *Firmicutes*, *Actinobacteria*, *Bacteroidetes* and *Proteobacteria*, and these phyla were also detected in the fermentation step, along with four additional phyla (*Chlorobi*, *Euryachacota*, *Fusobacteria* and *Nitrospira*). Analysis of the relative abundance of bacterial phyla level throughout the entire douchi fermentation process revealed that *Firmicutes*, *Actinobacteria* and *Proteobacteria* were the predominant phyla in both koji making and fermentation (Fig 1a), and this finding is consistent with previous reports [17,37]. Similarly, *Ascomycota* and *Zygomycota* were the dominant fungal phyla, as was observed in previous studies [18,38]. Indeed, the majority (> 40%) of OTUs in koji making were *Firmicutes*, and this did not change significantly throughout the koji making step. In contrast, the proportion of *Firmicutes* declined sharply during fermentation, from 69.55% (day 1) to 24.76% (day 15). *Proteobacteria* remained rare throughout koji making, and decreased from 0.13% (day 1) to 0.01% (day 7), but during the subsequent fermentation, *Proteobacteria* increased sharply, from 0.08% (day 1) to 46.57% (day 15), by which time they had become a dominant genus. *Actinobacteria* increased during koji making and fermentation, from an initial relative abundance of 3.28% (day 1) to 19.89% (day 12). Interestingly, *Actinobacteria* then declined to 9.67% by day 15. The proportion of *Firmicutes* and *Actinobacteria* remained stable in koji making and the early stages of fermentation, while *Proteobacteria* was barely detectable in koji making but became a dominant genus in fermentation, suggesting *Firmicutes* and *Actinobacteria* adapted to the koji making and fermentation environment. The observed decrease in the later stages of fermentation might be due to interspecific completion with *Proteobacteria* that underwent a sharp increase to 46.57% on day 15, at that same time that the other two dominant phyla decreased markedly. *Bacteroidetes*, *Chorobi*, *Euryachaeota*, *Fusobacteria* and *Nitrospira* phyla were barely detectable in koji making, and only slightly more abundant during fermentation.

At the genus level, 17 bacterial genera were detected during koji making, and 20 were detected during fermentation (Fig 1b). *Staphylococcus* and *Weissella* were the predominant genera in both steps, and constituted 42.59% and 9.81% of all bacterial sequences detected during the complete douchi fermentation process, respectively, consistent with previous studies [39–41]. *Staphylococcus* accounted for between 43.04% and 73.81% of genera present in koji making, and this genus declined dramatically during fermentation, from an initial 30.33% on day 1 to only 4.06% on day 3. Interestingly, this genus then recovered rapidly to reach 60.83% on day 9, but decreased sharply again to 9.75% by day 15. During koji making, the relative abundance of the other dominant genus, *Weissella*, declined sharply from an initial value of 1.75% to 0.26% on day 3, then increased rapidly to 7.57%. During fermentation, *Weissella* showed a similar trend to *Staphylococcus*, with the proportion increasing slightly from 34.05% to 48.79% between day 1 and 3. Interestingly, this genus then declined dramatically to 2.6%, but recovered to 15% by day 9, before continually declining to become a minority genus by the end of fermentation. Other major genera present in the koji making step included *Brevibacterium* and *Nocardiopsis*, each constituting more than 1% of total bacteria in this population. *Acinetobacter*, *Bacillus*, *Corynebacterium*, *Lactobacillus* and *Pseudomonas* were relatively abundant genera (>1%) during fermentation. The dynamics of the other genera in koji making and fermentation are shown in Fig 1b. In koji making, *Staphylococcus* was the dominating genus and maintained stably high levels, suggesting it is an aerobic or facultative anaerobic bacteria adapted to the koji making environment. *Staphylococcus* might play an important role in koji making, consistent with previous studies that revealed a role for *Staphylococcus gallinarum* in antibiotic activity and proteolytic capacity in the douchi fermentation process [42–44], possibly by providing a variety of amino acids or peptides for other genera to use during fermentation. Other major genera such as *Brevibacterium* and *Nocardiopsis* increased gradually and reached a maximum on day 7 in koji making, which indicated that these genera might play an important role during the later stages of koji making, consistent with the report that *Brevibacterium lactofermentum* produces glutamic acid [45]. Ganesh *et al*. reported that *Nocardiopsis* sp. can produce cellulolytic enzymes that enhance the flavour of douchi by degrading fiber into glucose [46]. Members of this genus also produce thermostable α-amylase [46].

During fermentation, *Staphylococcus* and *Weissella* remained the predominant genera, as was concluded in previous studies [40,47,48]. The proportion of *Staphylococcus* sharply decreased to 4.06% on day 3, before recovering to 40.44% then remaining a dominant genus, while the proportion of *Weissella* reached 48.79% but then decreased to 2.6% to become a minority genus (<1%). This indicates interspecific competition between *Staphylococcus* and *Weissella*. Ndagano *et al*. found that *Weissella* is activated by LAB in fermented food [49], and the metabolism of members of this genus, specifically that related to bacteriocins and acids, not only contributed to the formation of flavour compounds, but also inhibited the growth of pathogens with a low acid tolerance [50]. Other major genera in the fermentation step were still thriving on day 15, while the proportion of *Staphylococcus* and *Weissella* was 9.75% and 0.87%, respectively, suggesting *Acinetobacter*, *Bacillus*, *Corynebacterium*, *Lactobacillus* and *Pseudomonas* might play an important role during the later stages of fermentation, and metabolites secreted from these organisms might inhibit the growth of *Staphylococcus* and *Weissella*. *Acinetobacter* and *Pseudomonas* genera are able to degrade biosurfactants and hydrocarbons [51]. Among the microbiota in cheese, *Lactobacillus* possess some capacity for the breakdown of proteins during the fermentation process [52], and members of this genus are believed to be responsible for the distinctive sour taste and rich flavour [53]. *Bacillus* species play a crucial role by secreting microbial inhibitors and plasminogen during the fermentation of soybean [1], and *Bacillus subtilis* can enhance the angiotensin converting enzyme inhibitory effect of soybeans, as well as the anthocyanin content and reducing activity [54]. *Bacillus subtilis* also enhances lipases, amylases, proteases, cellulases and glutaminases that degrade lipids, proteins carbohydrates and flavonoid glycosides during the douchi fermentation process, resulting in metabolites such as organic acids, amino acids and aglycones that contribute to the taste, flavour, functionality and nutritional content [8].

### Comparison of bacterial community structure during koji making and fermentation

**Fig 2:**
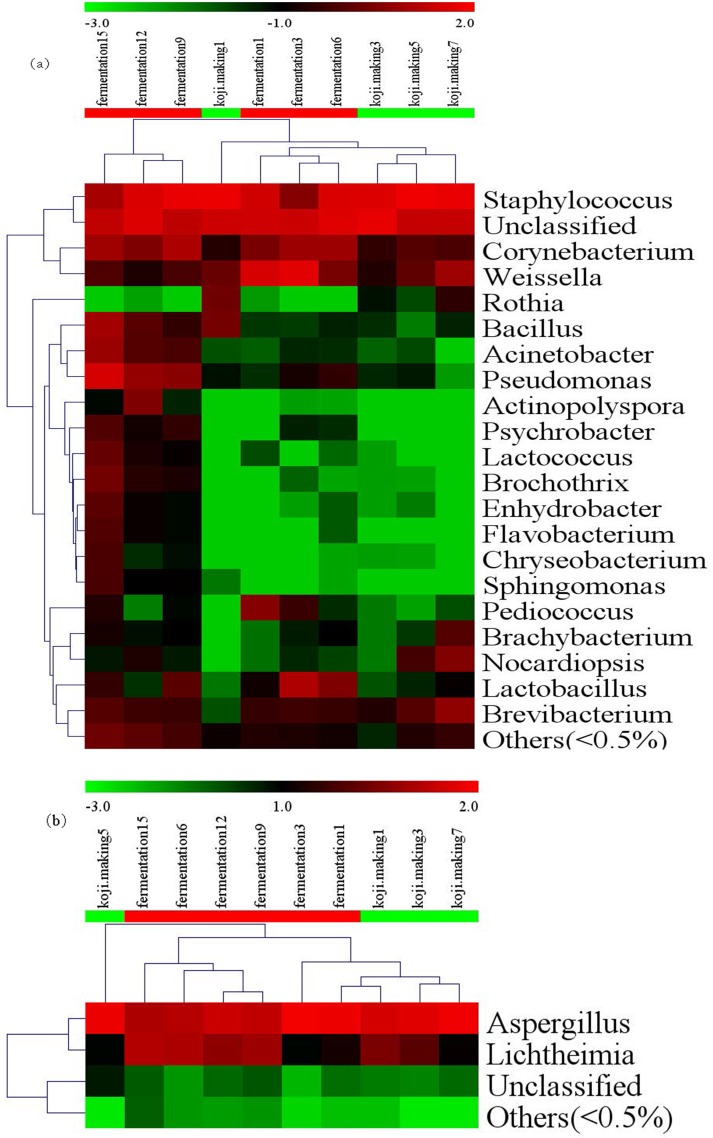
Heatmap and dendrogram of abundant bacterial (a) and fungal (b) genera present in the microbial community of 10 samples from koji making and fermentation. The heatmap plot indicates the relative abundance of genera in different samples (variables clustered on the vertical axis). The phylogenetic tree was calculated using the neighbour-joining method. The colour intensity is proportional to the relative abundance of bacterial and fungal genera.

**Fig 3:**
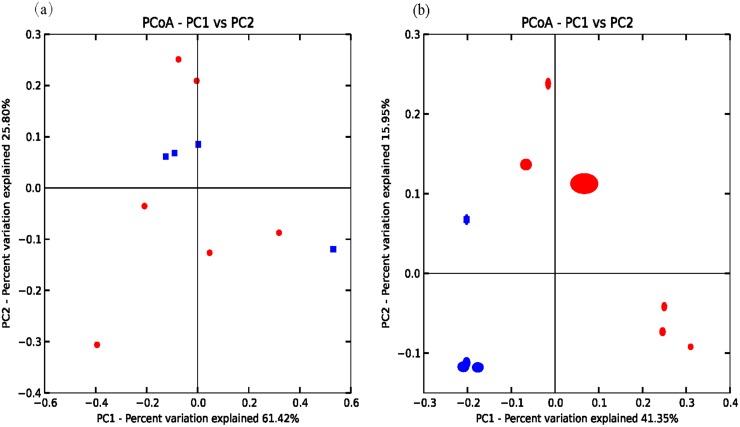
Principal coordinate analysis of microbial communities based on (a) weighted and (b) unweighted UniFrac metrics of samples from koji making and fermentation. Red and blue symbols represent samples from fermentation and koji making, respectively.

**Fig 4:**
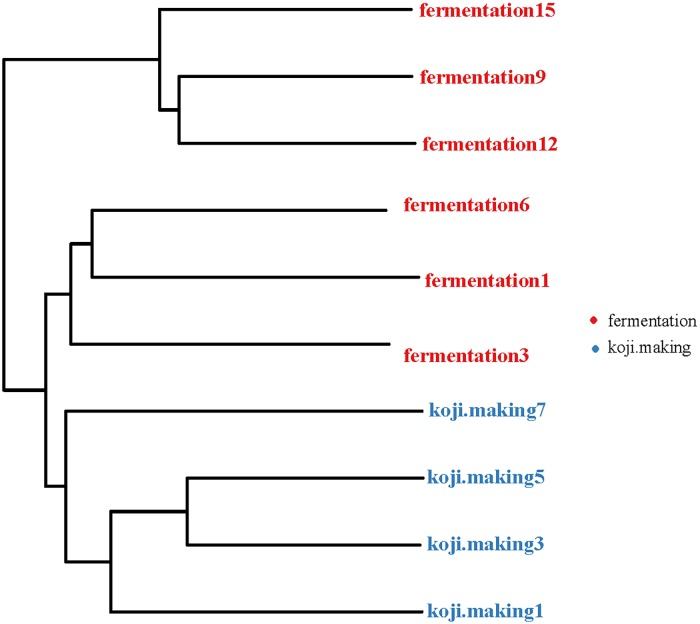
Cluster analysis of the bacterial microbiota in koji making and fermentation based on unweighted UniFrac distances.

A heatmap revealed differences in genera between koji making and fermentation (Fig 2a), and PCoA and cluster analyses were performed to evaluate similarities in bacterial communities in the two stages. Both weighted (PC1 variance = 61.42%, PC2 variance = 25.80%; Fig 3a) and unweighted (PC1 variance = 41.35%, PC2 variance = 15.95%; Fig 3b) PCoA was performed. UniFrac values revealed an overlap in bacterial communities between koji making and fermentation, but multivariate analysis of variance indicated a clear, significant difference (p < 0.05) between koji making and fermentation based on the principal coordinates of the unweighted UniFrac metric. Unweighted pair-group analysis (UPGMA) using arithmetic means (Fig 4) based on unweighted UniFrac analysis also indicated a discriminative structural separation between steps, and furthermore, the bacterial communities were relatively stable during koji making, but fluctuated considerably during fermentation from day 1 to 6, and day 9 to 15.

**Fig 5:**
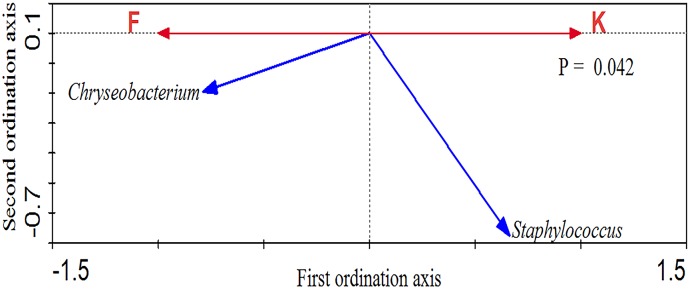
Bioplot of redundancy analysis of bacteria in koji making and fermentation. F represents fermentation and K represents koji making.

Redundancy analysis (RDA) was performed using koji making or fermentation as constrained explanatory variables and the relative abundance of all OTUs as the response variable. The significance of RDA was confirmed by a Monte Carlo Permutation test (p = 0.042; Fig 5) and 26% of the variance in OTU abundance data could be explained by the canonical axis. Two key responding OTUs were identified that were well correlated with sample scores on the canonical axis, for which at least 26% of the variability was explained by the axis (Fig 5). In the RDA ordination plot, one OTU (*Staphylococcus*) was enriched in koji making, while one OTU (*Corynebacterium*) was enriched in fermentation. Differences in microbiota could reflect differences in the environments of the two stages, and indicate a marked transition between steps [55,36,56]. In this regard, Montal *et al*. reported that environmental factors such as the amount of oxygen, and the time of inoculation (e.g. during the ripening process) are of great importance [57].

### Fungal communities present in koji making and fermentation stages of douchi fermentation

**Fig 6:**
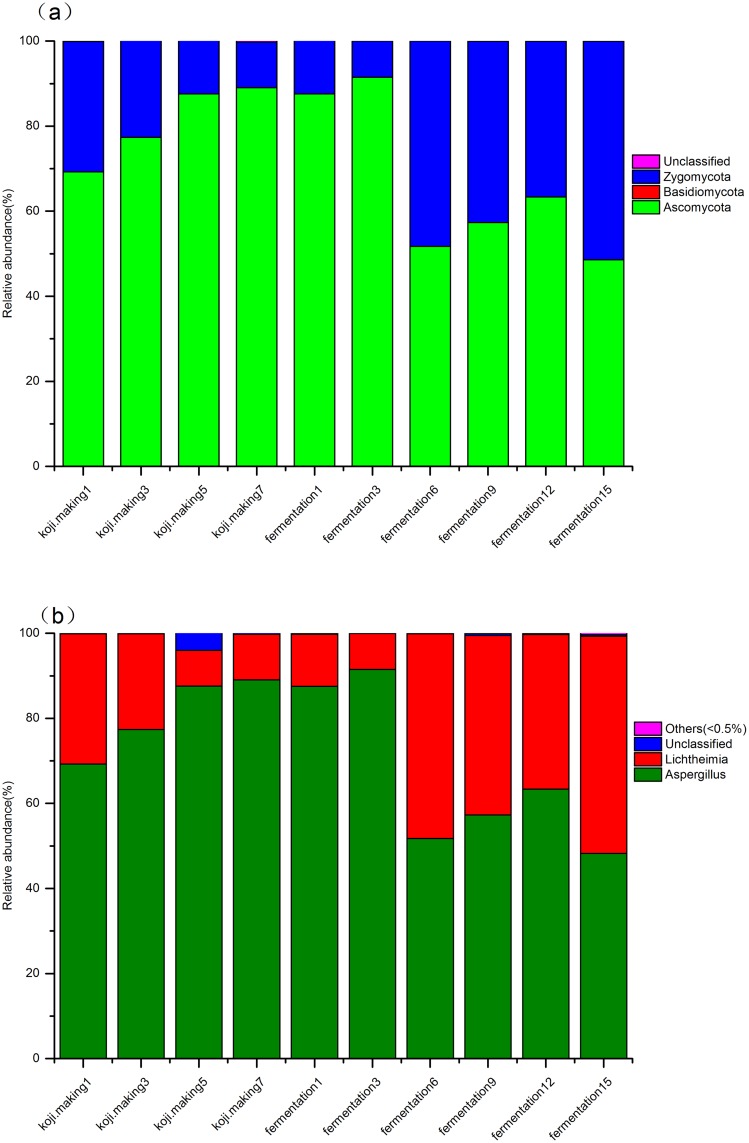
Relative abundance of fungi at phyla (a) and genus (b) levels during koji making and fermentation.

Compared with bacteria, the overall diversity of the fungal microbiota in douchi fermentation was lower. Only two and three fungal phyla were detected in koji making and fermentation, respectively. *Ascomycota* and *Zygomycota* were detected in both steps of the process, and the relative abundance of fungi at the phylum level (Fig 6a) showed that *Ascomycota* were dominant, accounting for 72.33%, while *Zygoycota* was the second most dominant phyla, accounting for 27.63%. *Ascomycota* and *Zygomycota* were the dominant fungal phyla, as was observed in previous studies [18,58]. *Ascomycota* and *Zygomycota* contributed 80.8% and 19.11% in koji making, and 66.68% and 33.31% in fermentation, respectively.

At the genus level, two genera, *Aspergillus* and *Lichtheimia*, dominated both koji making and fermentation, accounting for 72.29% and 27.09%, respectively (Fig 6b). *Aspergillus* dominated throughout koji making, increasing gradually from 69.24% on day 1 to 89.07% on day 7, and this genus continued to increase during fermentation, reaching 91.48% on day 3. However, this genus then declined to 51.76% on day 6 of fermentation, before increasing gradually to 63.33% on day 12, then decreasing to 48.26% by the end of the process. *Lichtheimia* was the second most abundant genus in koji making, during which members decreased from 30.63% on day 1 to 10.72% on day 7. In the early stages of fermentation, the percentage of *Lichtheimia* declined gradually from 12.26% on day 1 to 8.50% on day 3, then increased dramatically to 48.16% before declining to 36.40% on day 12, and finally recovering to 51.05% on day 15 to become the dominant genus at the end of the process. These two predominant genera might play an important role over the whole fermentation process. Kim *et al*. reported that *Aspergillus oryzae* secretes a large quantity of amylases and/or proteases that break down starches and proteins into sugars and peptides/amino acids that are then absorbed and utilized by yeasts and lactic acid bacteria in the fermentation stage [59]. *Aspergillus oryzae* may therefore play a key role in providing a suitable living habitat for fermentative microbes that are essential during the second step of the process [56]. Although *Aspergillus* did decline during fermentation, from a maximum of 91.48% on day 3 to 48.26% on day 15, *Aspergillus oryzae* remained a major genus throughout fermentation, indicating a high tolerance to the dry, high salt and high temperature conditions of the second step. *Lichtheimia* gradually declined in abundance during koji making, but increased during fermentation, from 12.26% on day 1 to 51.05% on day 15, suggesting this genus was adapted to the harsh environment, and indicating a key role in douchi fermentation [56], consistent with previous studies [60,61]. Burgess *et al*. reported that *Lichtheimia* can survive at temperatures of 50–60°C [62], while Garcia *et al*. found that *Lichtheimia* produce rhamnose from β-glucosidase activity during solid-state fermentation[63], which leads to the release glucose that may improve the flavour of douchi. Filamentous fungi are usually included in solid state fermentation processes due to their high production of hydrolytic enzymes and relatively high tolerance to low water activity [64].

### Comparison of fungal community structure during koji making and fermentation

**Fig 7:**
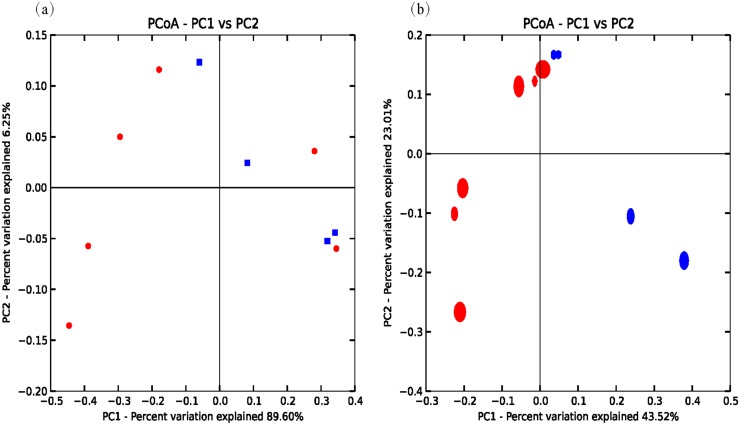
Principal coordinate analysis of microbial communities based on (a) weighted and (b) unweighted UniFrac metrics of samples from koji making and fermentation. Red and blue symbols represent samples from fermentation and koji making, respectively.

**Fig 8:**
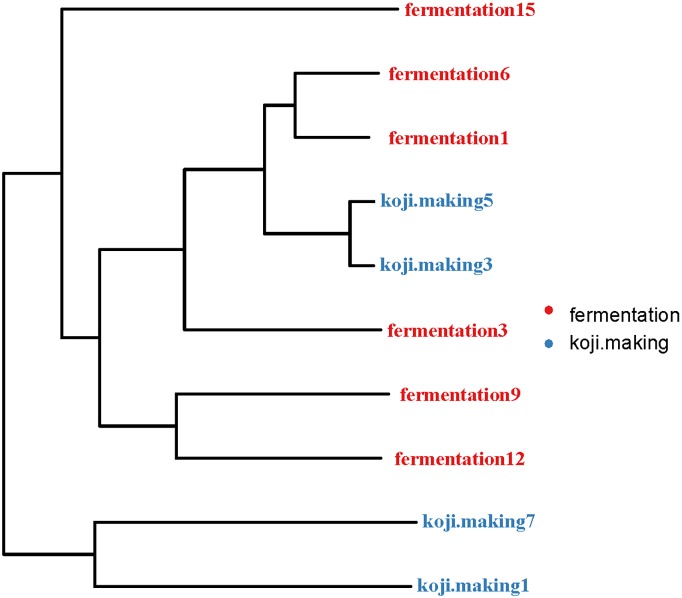
Cluster analysis of fungal microbiota in koji making and fermentation based on unweighted UniFrac distances.

Multivariate analysis of variance of the fungal microbiota present in koji making and fermentation steps was performed using both weighted (PC1 variance = 89.6%, PC2 variance = 6.25%; Fig 7a) and unweighted (PC1 = 43.52%, PC2 = 23.01%; Fig 7b) parameters. UniFrac PCoA based on fungal microbiota communities revealed no distinct clustering pattern between koji making and fermentation (Fig 8). Similarly, multivariate analysis of variance based on unweighted UniFrac distances also indicated that there were no significant differences (p > 0.05) between fungal communities during koji making and fermentation. However, heatmap analysis did indicate differences in genera between steps, and non-parametric Mann—Whitney tests confirmed that no genera showed a significant difference (p < 0.05) between koji making and fermentation (Fig 2b).

## Conclusions

In this study, bacterial and fungal diversity in the douchi fermentation process was studied by high-throughput sequencing to investigate the dynamics of the dominant genera and phyla, and to identify microorganisms that contribute to the distinct flavour and character of this traditional fermented food. This is the first study to apply this technology to studying douchi fermentation. The results provide insight into the dynamics of microbial community diversity that could be useful for improving the industrial production of douchi, and ensuring that high quality and safety are maintained.

## Supporting Information

S1 Fig Rarefaction cure for (a) observed species and (b) Simpson diversity of bacteria. (TIF)

S2 Fig Rarefaction cure for (a) observed species and (b) Simpson diversity of fungi. (TIF)

S1 Table Comparison of bacterial alpha diversity. (DOCX)

S2 Table Comparison of fungal diversity. (DOCX)
