# Supplementary figures and images for: High-Throughput Sequencing of Microbial Community Diversity and Dynamics during Douchi Fermentation
Source: PLoS One. 2016 Dec 19;11(12):e0168166. doi: 10.1371/journal.pone.0168166 (PMC5167551; doi:10.1371/journal.pone.0168166)

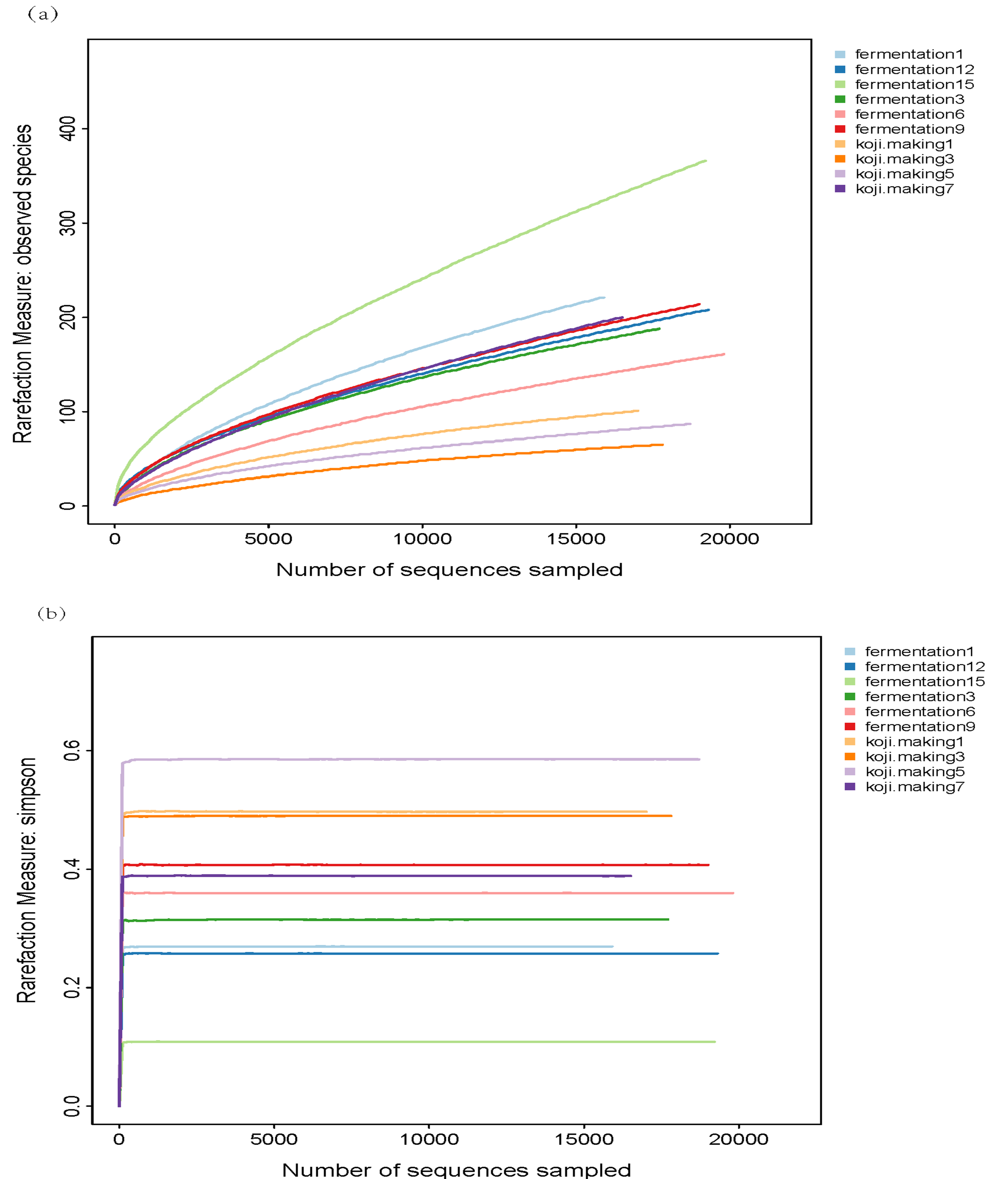

Supplement: S1 Fig — (TIF) [file pone.0168166.s001.tif]

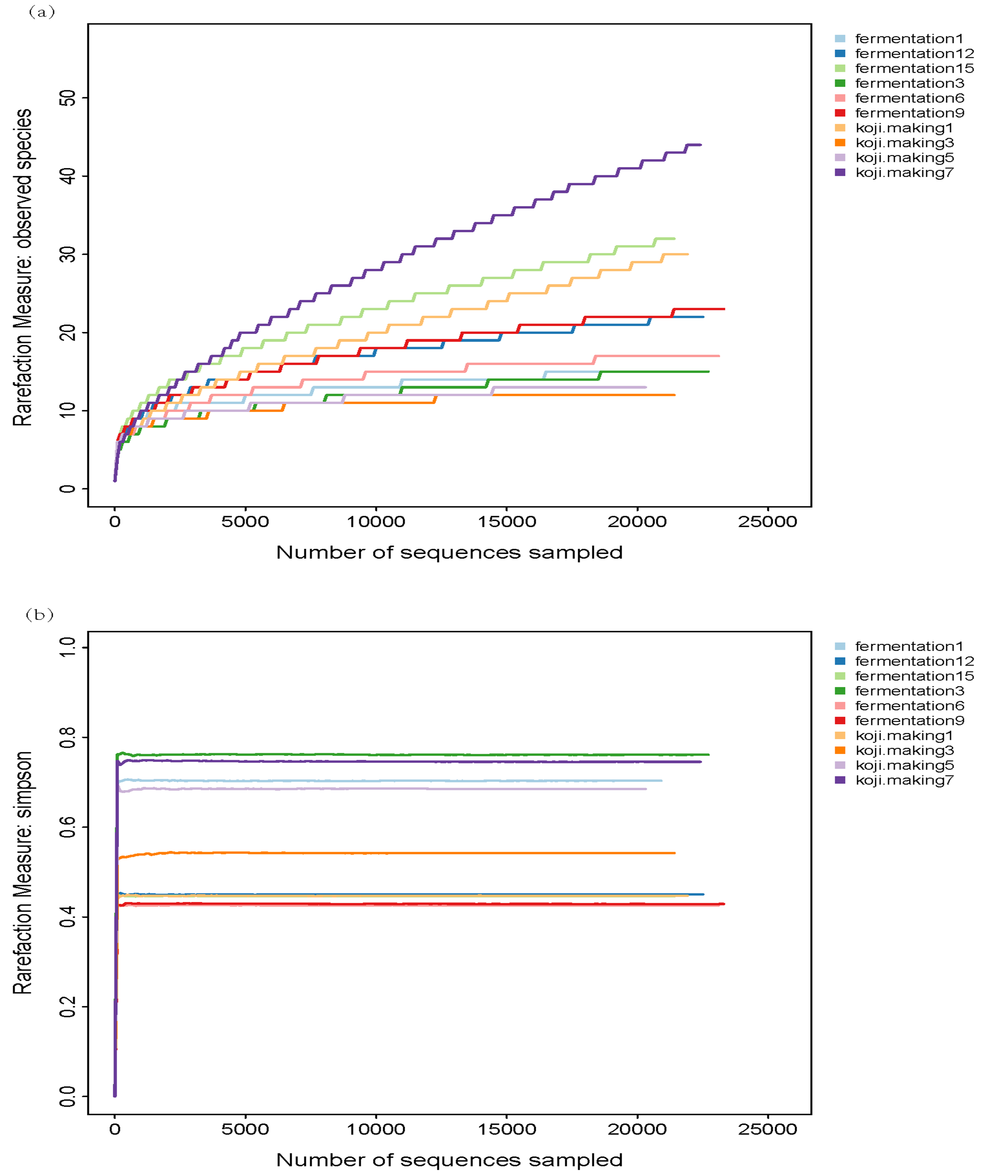

Supplement: S2 Fig — (TIF) [file pone.0168166.s002.tif]
