# Supplementary material for: High-Throughput Sequencing of Microbial Community Diversity and Dynamics during Douchi Fermentation
Source: PLoS One. 2016 Dec 19;11(12):e0168166. doi: 10.1371/journal.pone.0168166 (PMC5167551; doi:10.1371/journal.pone.0168166)
Supplement: S1 Table — (DOCX) [file pone.0168166.s003.docx]

Table S1. Comparison of the bacterial alpha diversity

| Diversity index | Mean (Koji making) | SD (Koji making) | Mean (Fermentation) | SD (Fermentation) | p-value |
| --- | --- | --- | --- | --- | --- |
| Sobs | 113.25 | 59.70134 | 226.6667 | 72.15446 | 0.0381 |
| Chao 1 | 253.0848 | 161.0017 | 605.2136 | 321.9529 | 0.06667 |
| Ace | 384.3733 | 314.9471 | 1023.086 | 561.7431 | 0.06667 |
| Shannon | 1.04374 | 0.30186 | 1.777592 | 0.50677 | 0.01905 |
| Simpson | 0.48999 | 0.08047 | 0.28627 | 0.10334 | 0.01905 |
