# Supplementary material for: High-Throughput Sequencing of Microbial Community Diversity and Dynamics during Douchi Fermentation
Source: PLoS One. 2016 Dec 19;11(12):e0168166. doi: 10.1371/journal.pone.0168166 (PMC5167551; doi:10.1371/journal.pone.0168166)
Supplement: S2 Table — (DOCX) [file pone.0168166.s004.docx]

| Diversity index | Mean (Koji making) | SD (Koji making) | Mean (Fermentation) | SD (Fermentation) | p-value |
| --- | --- | --- | --- | --- | --- |
| Sobs | 24.75 | 15.26161 | 20.6667 | 6.53197 | 0.91485 |
| Chao 1 | 52.71429 | 46.09042 | 30.26667 | 19.31027 | 1 |
| Ace | 90.46288 | 92.02207 | 31.92929 | 18.3291 | 1 |
| Shannon | 0.8274 | 0.21851 | 0.88874 | 0.20665 | 0.91429 |
| Simpson | 0.60504 | 0.13545 | 0.53591 | 0.15349 | 0.47619 |

Table S2. Comparison of the fungal diversity
